# Variation of VP2 stoichiometry and deamidation of VP1 during production and their impacts on the transduction efficiency of AAV vectors

**DOI:** 10.1016/j.omtm.2025.101581

**Published:** 2025-09-01

**Authors:** Takahiro Maruno, Mitsuko Fukuhara, Yasuo Tsunaka, Aoba Matsushita, Kiichi Hirohata, Karin Bandoh, Megumi Onaka, Risa Shibuya, Yuki Yamaguchi, Haruka Nishiumi, Yoshiki Nagashima, Daisuke Higo, Toshie Kuwahara, Tomoko Ueno, Masaharu Maeda, Guirong Kanai-Bai, Noriko Yamano-Adachi, Tetsuo Torisu, Takeshi Omasa, Susumu Uchiyama

**Affiliations:** 1 U-Medico Inc., 2-1 Yamadaoka, Suita, Osaka 565-0871, Japan; 2 Graduate School of Engineering, The University of Osaka, 2-1 Yamadaoka, Suita, Osaka 565-0871, Japan; 3 Thermo Fisher Scientific, 3-9 Moriya-cho, Kanagawa-ku, Yokohama, Kanagawa 221-0022, Japan; 4 Osaka Consolidated Laboratory, Manufacturing Technology Association of Biologics, 2-1 Yamadaoka, Suita, Osaka 565-0871, Japan

**Keywords:** gene therapy, adeno-associated virus vector, AAV vector, vector manufacturing, viral protein stoichiometry, deamidation, transduction efficiency, physicochemical properties

## Abstract

Recombinant adeno-associated viruses (rAAVs) are a leading platform for *in vivo* gene therapy. However, limited information is available on the fluctuation of quality attributes (QAs), during manufacturing using the HEK293 suspension cell line system, which is suitable for small- to large-scale production using bioreactors. In this study, we evaluated 14 QAs and their variations, including the viral protein (VP) stoichiometric ratio and deamidation ratios of asparagine residues, of the products for AAV8 with enhanced green fluorescent protein as a gene of interest, which were produced employing essentially the same production procedures with slight modification in the upstream conditions. The VP stoichiometric ratios of the manufactured adeno-associated virus (AAV) vectors differed significantly among the products. Correlation analysis of the QAs revealed the attributes that could affect the transduction efficiency. Notably, the VP2 stoichiometric ratio was positively correlated with the transduction efficiency. The deamidation ratios of N57 and N94 in VP1 had a negative tendency to the transduction efficiency. Consistently, the accelerated stability testing showed a negative correlation of the time-dependent increase in the deamidation ratios of the two asparagine residues with the transduction efficiency. This study highlights the importance and implications of the examined QAs in the development of rAAVs.

## Graphical abstract

**Figure:**
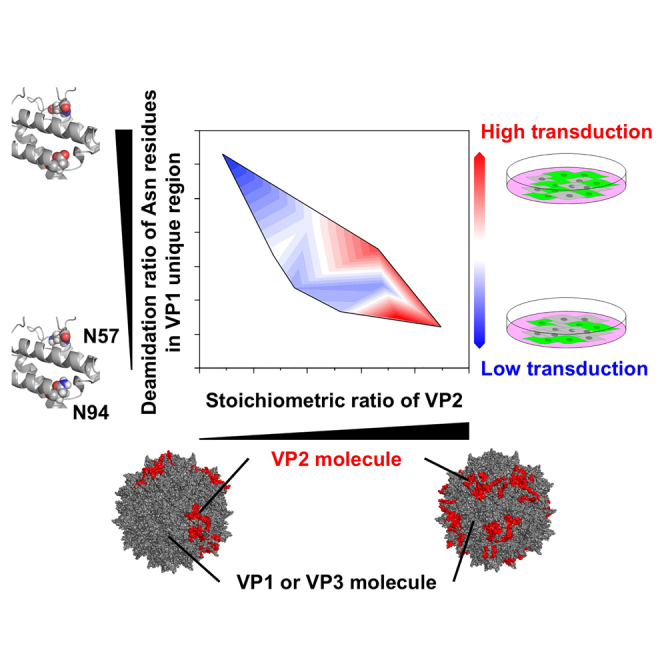

## Teaser

Uchiyama and colleagues demonstrated, based on the repeated productions of adeno-associated virus vector using HEK293 suspension cell line, that the stoichiometric ratio of viral protein 2 and deamidation ratios of Asn57 and Asn94 in viral protein 1 could be potential critical quality attributes (pCQAs) in terms of gene transduction.

## Introduction

Adeno-associated viruses (AAVs) contain a protein capsid outer coat approximately 25 nm in diameter with an icosahedral structure and a viral genome.^1^^,^^2^ AAV vectors have been developed for gene therapy, and 8 AAV vector drugs have been successfully marketed by the end of 2024. Thus, AAV vectors are a leading platform for *in vivo* gene therapy. The capsid of an AAV vector consists of three viral capsid proteins,^3^ VP1, VP2, and VP3, and DNA up to approximately 5 kb long is encapsidated in the capsid. The amino acid sequence of VP3 is identical to that of the VP3 region in VP1 and VP2.^3^ VP2 has an extended region at the N-terminus of VP3. VP1 has an extended region at the N-terminus of VP2.^3^ VP3 variants are produced depending on the initiation codon at the translation initiation site.^4^ The unique region of VP1 plays an important role in endosomal escape, while the VP1/VP2 common region functions in nuclear transfer.^5^^,^^6^ However, the unique and common VP1/VP2 regions are not essential for capsid formation, as opposed to the shared VP3 region.^7^ In fact, expression of only VP3 can produce authentic icosahedral AAV capsids composed of VP3.^8^ The typical ratio of VP1:VP2:VP3 is approximately 1:1:10.^9^^,^^10^ Since an icosahedral capsid is composed of 60 VP molecules, its stoichiometric ratio is approximately 5:5:50.

The VP stoichiometric ratio is a significant quality attribute (QA) that requires evaluation. In the upstream process of AAV vector manufacturing, HEK293 cells, HEK293 cell-derived cells, such as adherent HEK293T cells or suspension HEK293 cells, are employed in the production system via the transient transfection of plasmids.^11^ To mitigate the potential risk, HEK293 cells without the T-antigen protein and/or T antigen-encoding nucleotide sequence^12^ are preferred for the production of AAV vectors. Alternative to transient transfections include adenovirus, herpesvirus, and vaccinia virus infection-based methods and producer cell lines for mammalian cell-based AAV vector production systems. The VP1 expression level was generally higher in the AAV vector produced using HEK293 production systems than those produced using baculovirus production systems.^13^^,^^14^ When the percentage of VP1 molecules to the total number of VP molecules in the baculovirus production systems was low, the transduction efficiency decreased.^15^ VP1-abundant AAV vectors exhibit high transduction efficiency *in vitro* and *in vivo*.^16^ Some studies have demonstrated that an insufficient VP1 reduces the rate of vector transduction.^13^^,^^15^^,^^17^^,^^18^^,^^19^ More recently, we established an accurate method for the quantitation of the VP stoichiometric ratio using capillary gel electrophoresis (CGE) and mass spectrometry.^4^^,^^20^ With the established method, comparison between low- and high-density AAV vectors produced using HEK293 production system revealed that the low-density AAV vectors with a higher stoichiometric ratio of VP1 and VP2 have higher transduction efficiency than the high-density AAV vectors having a lower ratio of VP1 and VP2 ratios *in vitro*.^20^ Thus, because the VP stoichiometric ratio affects the transduction efficiency regardless of the expression system, the VP stoichiometric ratio of each product must be accurately evaluated. Notably, a previous study reported that VP2 is not essential for transduction efficiency.^7^

Other QAs that have been discussed as potential factors affecting the transduction efficiency include the full-empty ratio^21^ and post-translational modifications.^22^ Empty capsid carries immunogenic risks, as well as the risk of reduced transduction efficiency. Because Recombinant adeno-associated viruses (rAAVs) are generally administered in gene therapy based on the number of viral genomes (VGs), even if the VG dose remains fixed, variations in the full-empty ratio alter the total number of capsid particles administered to the patient. This could lead to significant variations in patient outcomes caused by the relationship between the capsid particle dose and immune response. In one study, mice were treated with AAV8 encoding the factor IX gene as a gene of interest (GOI) and its empty by-product. The administration of empty particles resulted in significant CD8^+^ T cell proliferation in the liver compared with the negative control, although not as effectively as full particles.^23^ Furthermore, empty particles competing with full particles for binding sites on target cells may reduce the transduction to the target cells. Adding empty particles to a fixed dose of AAV8 vectors containing a liver-targeted GOI inhibited transduction to the liver.^21^ The European Medicines Agency, US Food and Drug Administration, and Pharmaceuticals and Medical Devices Agency have listed the number of empty particles or the full-empty ratio as one of the evaluation points in the respective guidelines issued by each agency.^24^^,^^25^^,^^26^

The deamidation of the capsid protein negatively affected the transduction efficiency *in vitro*^22^ and *in vivo*^27^ and is listed as a potential critical quality attribute (pCQA).^28^ Deamidation in AAVs tends to occur at asparagine (Asn, N) residues especially when the adjacent residue is glycine (Gly, G), which is a well-known QA used in the quality assessment of therapeutic antibodies. The focus was on seven residues (N57, N94, N263, N305, Q467, N479, and N653) that cause a more than 10-fold reduction in the transduction efficiency of the AAV8 vector based on experiments using the Asn residue to substitute the aspartic acid residue or the glutamine residue to substitute the glutamic acid residue.^23^ Furthermore, three residues (N57, N329, and N514) of the AAV8 capsid protein and four residues (N57, N329, N452, and N512) of the AAV9 capsid protein could be involved in immune responses, as reported previously.^29^ Monitoring these QAs is essential to ensure the supply of safe and therapeutically effective AAV vectors. Moreover, the extent of variation in the QAs of each manufactured product with different manufacturing lots and conditions and its correlation with variation in other QA parameters needs to be elucidated.

Although several QAs of AAV vectors that were potential pCQAs have been reported, information is limited on the variations in QAs of the AAV vector product under similar production and purification procedures and the corresponding effects on the transduction efficiency of the produced AAV vector. Therefore, in this study, we manufactured 10 AAV vector products under the conditions, with the only differences being the culture volume and container type. The products were then purified through the same purification procedure and evaluated for variations in QAs. Fourteen QAs of all products were examined, including particle properties, particle size distribution, viral protein properties, impurities, and transduction efficiency. Correlation analysis of acquired QAs was then performed. The purified AAV vector was subjected to short-term accelerated stability testing, stored at 25°C, and assessed for variations in the deamidation ratio and transduction efficiency that could occur during storage.

In addition, we evaluated several parameters by monitoring the purification process and examining their relationships to the QAs of the products. Specifically, information on the affinity chromatography (AC) peak area, absorbance at 260 nm (A260)/absorbance at 280 nm (A280) ratio after AC, density gradient ultracentrifugation (DG-UC) peak area, and A260/A280 ratio after DG-UC for the downstream process were obtained.

We succeeded in extracting QAs that affected transduction efficiency based on the QA assessments and correlation analysis of the obtained parameters for AAV vectors produced employing essentially the same production procedures with slight modification in the upstream conditions. Further assessment regarding the impact of QAs on the transduction efficiency was carried out by using AAV vectors prepared by short-term accelerated stability testing. These results helped to clarify the points considered in the future production of AAV vectors.

## Results

### AAV vector manufacturing

**Figure 1:**
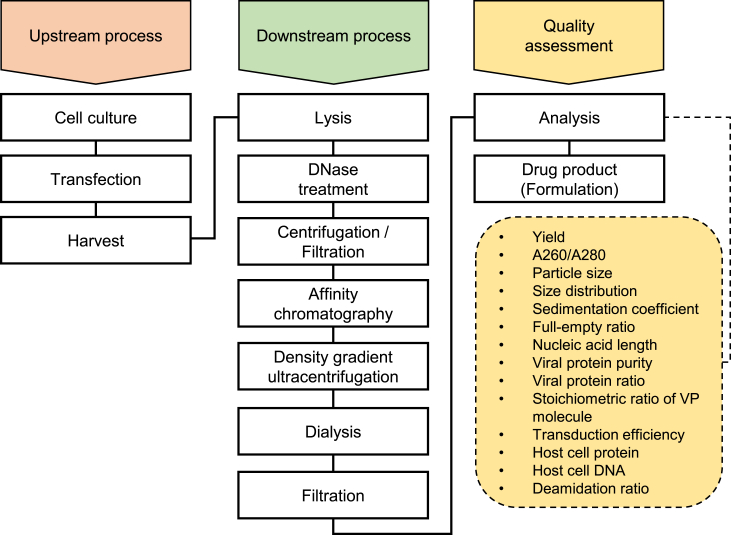
Upstream and downstream processes for 10 adeno-associated virus vectors that have the cytomegalovirus promoter and enhanced green fluorescent protein (AAV8-CMV-EGFP) production and quality assessment The processes in the upstream, downstream, and quality assessment processes. To clarify, processes are colored in orange (upstream process), green (downstream process), and yellow (quality assessment).

The variations in the QAs of AAV vectors with the same serotype and GOI manufactured under the same conditions, except for culture volume and container type, were evaluated by manufacturing AAV vectors that contained the cytomegalovirus (CMV) promoter and enhanced green fluorescent protein (EGFP) as the GOI (AAV8-CMV-EGFP). The conditions are summarized in Table 1, and the manufacturing workflow is shown in Figure 1. The mean (±standard deviation [SD]) yield of the final product was 4.4 × 10^13^ ± 2.2 × 10^13^ vg per 0.2 L of culture volume. The mean (±SD) yield was 2.7 × 10^13^ ± 1.8 × 10^13^ vg for batch A, 4.5 × 10^13^ ± 0.3 × 10^13^ vg for batch B, and 6.6 × 10^13^ ± 1.8 × 10^13^ vg for batch C. The in-process results for the downstream process, including the peak areas of the AC and DC-UC, yield, and A260/A280 ratio at each purification step, are shown in Figures S1 and S2.

**Table 1:** Summary of conditions in the upstream process

Product No.	Batch	Culture	Culture volume (L)	Transfection reagent	Harvest after transfection (days)	Purified volume (L)
1	A	1 L flask	0.2	FectoVIR-AAV	4	0.2
2						
3						
4						
5	B	2 L flask	1			
6						
7						
8	C	2 L bioreactor				
9						
10

### QAs of the AAV8-CMV-EGFP

To extract the parameters that varied among the AAV vectors, we evaluated 14 QAs of the 10 AAV8-CMV-EGFP vectors. The results of each QA are described as follows.

#### Capsid integrity

**Figure 2:**
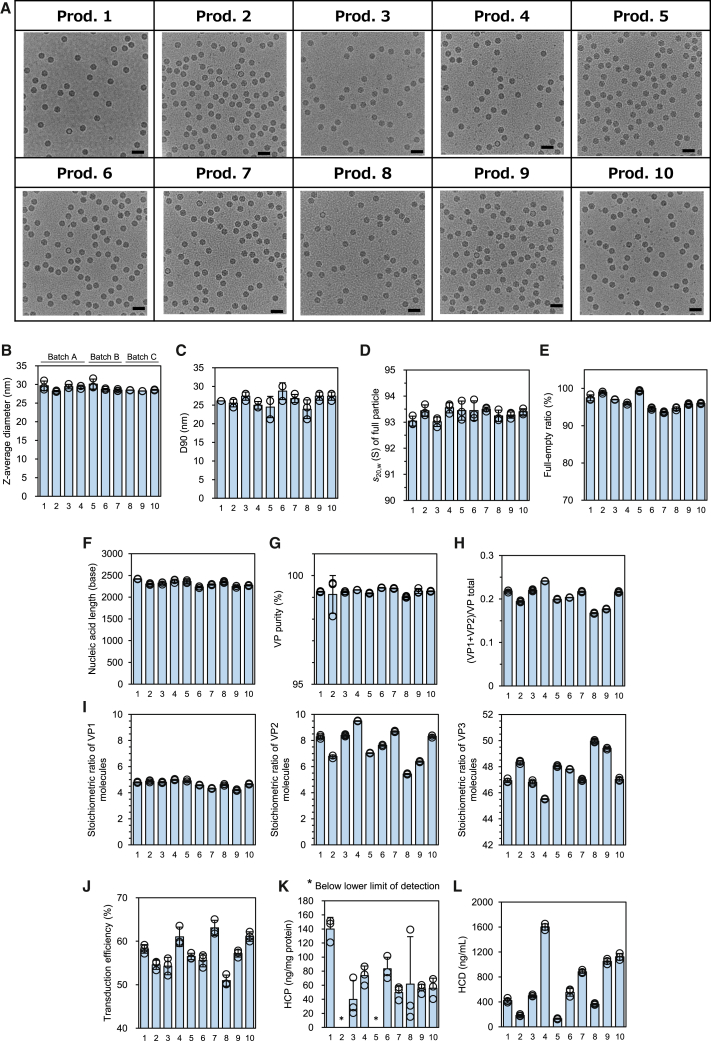
Capsid integrity, quality attributes, and product-to-product variation in 10 AAV8-CMV-EGFP products (A) Cryo-electron micrographs of 10 AAV8-CMV-EGFP products. Scale bars: 50 nm. (B–L) Bar graphs showing the parameters of the 10 products of each quality attribute (QA). The plots show the results of each measurement, and the bars show the mean values obtained from triplicate experiments. Error bars indicate standard deviation (SD). The mean and SD values were calculated from experiments performed in triplicate on the QA of each AAV vector product and used as the parameter for each product. (B) Z-average diameter. (C) D90. (D) Sedimentation coefficient, *s*_20,w_, of full particle. (E) Full-empty ratio. (F) Nucleic acid length. (G) Viral protein (VP) purity. (H) (VP1+VP2)/VP_total_. (I) Product-to-product variation in the stoichiometric ratio of VP molecules, VP1 (left), VP2 (middle), and VP3 (right). (J) Transduction efficiency. (K) Host cell protein (HCP). (L) Host cell DNA (HCD). The numbers on the horizontal axis indicate product numbers.

The 10 final products were examined for capsid integrity by cryoelectron microscopy (cryo-EM). Genome-filled and empty particles were observed, similar to electron micrograph images obtained in a previous study.^30^ In all the products manufactured in this study, the AAV vector particles successfully formed particles (Figure 2A). Furthermore, most of the particles observed in all products were full particles, and no assemblies of particles that might have been aggregates were found. The full-empty ratio of each product was estimated using a convolutional neural network (CNN)-based image classification method developed in our laboratory^31^ and ranged from 91.2% (Prod. 8) to 98.2% (Prod. 5; Table S1). Next, we examined the 14 QAs of the AAV8-CMV-EGFP of these 10 products.

#### Particle properties and distributions

The Z-average diameter of the main components observed in the dynamic light scattering (DLS) analysis were also examined. The mean Z-average diameter of each product obtained by repeated experiments ranged from 28.2 nm (Prod. 2) to 30.1 nm (Prod. 5) for the 10 products (Figure 2B), which is in good agreement with those of commonly accepted AAV vectors.^32^^,^^33^^,^^34^^,^^35^ D90, an indicator less influenced by aggregates than the mean Z-average diameter, was used to evaluate the size distribution of the product. The mean D90 of each product obtained through replicate experiments ranged from 23.9 nm (Prod. 8) to 28.7 nm (Prod. 6) for the 10 products (Figure 2C). The small variation in size distribution could be originated from the uniformity of the purification method and final buffer conditions. The values were also in good agreement with the lack of aggregates observed on cryo-EM experiments (Figure 2A).

The mean sedimentation coefficient (*s*_20,w_) of the full particle of each product obtained from repeated experiments ranged from 93.0 S (Prod. 1 and 3) to 93.6 S (Prod. 4) for the 10 products (Figure 2D). The sedimentation coefficient distribution of each product demonstrated the high purity of the manufactured AAV8 vectors (Figure S3). The estimated nucleic acid length from *s*_20,w_, calculated using Equation 1 (see materials and methods), ranged from 2.56 kb (Prod. 1 and 3) to 2.61 kb (Prod. 4) and agreed well with the designated nucleic acid length of 2.52 kb. The particle concentrations of both full and empty particles were determined by calculating each peak area from the sedimentation coefficient distribution profile and the molar extinction coefficient (*ε*) of each species at a detection wavelength of 230 nm obtained by band sedimentation analytical ultracentrifugation (BS-AUC). The mean full-empty ratio, F/(E + F), of each product obtained by repeated experiments ranged from 93.6% (Prod. 7) to 99.4% (Prod. 5) for the 10 products (Figure 2E). The full-empty ratio obtained by BS-AUC was well correlated with the values estimated by CNN-based image classification of cryo-EM images (Figure S4).

#### Length of the main nucleic acid component

The length of the primary nucleic acid component extracted from the product was evaluated by CGE (Figure S5). The lengths of the nucleic acids of the 10 products ranged from 2.2 kb (Prod. 6) to 2.4 kb (Prod. 1) (Figure 2F). Compared with the designated nucleic acid length of 2.52 kb, the mean length obtained here was approximately 8% shorter, which is presumably due to inverted terminal repeats (ITRs) at both ends of the DNA, forming a secondary structure that could induce genomic DNA compaction.

#### Viral protein properties

VP assessments were performed using CGE. The mean VP purity of each product obtained from repeated experiments ranged from 99.0% (Prod. 8) to 99.4% (Prod. 6 and 7) for 10 products (Figure 2G). The downstream process employed in this study can remove a high proportion of protein contaminants. The numbers of VP1, VP2, and VP3 molecules in 60 VP molecules per AAV vector particle were estimated (Figure S6). The mean ratio of (VP1 + VP2)/VP_total_ is positively correlated with AAV vector activity.^20^ In this study, the value of (VP1 + VP2)/VP_total_ of each product, which was obtained by repeated experiments, ranged from 0.167 (Prod. 8) to 0.241 (Prod. 4) for the 10 products (Figure 2H). The variations in the (VP1 + VP2)/VP_total_ was attributed to changes in the stoichiometric ratio of the VP2 molecule; the mean (±SD) stoichiometric ratios of the VP1, VP2, and VP3 molecules were 4.7 (±0.3), 7.6 (±1.2), and 47.7 (±1.3), respectively. Figure 2I clearly shows that the stoichiometric ratio of VP molecules changed with the differences in manufacturing conditions. The highest stoichiometric ratio obtained was 9.5 for Prod. 4, while the lowest stoichiometric ratio of VP2 molecules observed was 5.4 for Prod. 8.

#### Transduction efficiency

At 50,000 multiplicity of infection (MOI), the transduction efficiency based on the percentage of GFP-positive cells of each product obtained by replicate experiments ranged from 50.9% (Prod. 8) to 63.1% (Prod. 7) for the 10 products (Figure 2J). The relationship between the transduction efficiency and MOIs is shown in Figure S7.

#### Host cell protein and host cell DNA

The mean host cell protein (HCP) content of each product obtained in replicate experiments ranged from below the detection limit to 139.8 ng/mg (Prod. 1) for the 10 products (Figure 2K). The highest HCP content (139.8 ng/mg) was observed in Prod. 1. The HCP contents of Prods. 2 and 5 were below the lower detection limit. The mean host cell DNA concentration of each product obtained via replicate experiments ranged from 130.6 ng/mL (Prod. 5) to 1,600.8 ng/mL (Prod. 4) for the 10 products (Figure 2L).

#### Deamidation ratios of capsid proteins

**Figure 3:**
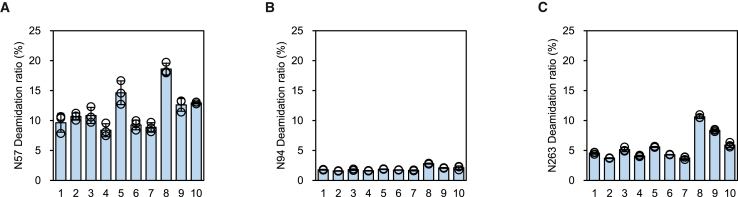
Deamidation ratios of N57, N94, and N263 in 10 AAV8-CMV-EGFP products (A–C) Bar graphs showing the parameters of the 10 products with different deamidation ratios. The plot shows the results of each measurement, and the bars indicate the mean values obtained from the experiments performed in triplicate. Error bars indicate standard deviation (SD). The mean and SD values were calculated from experiments performed in triplicate for each AAV vector product and used as the parameter for each product. The numbers on the horizontal axis indicate product numbers.

Nine residues (N57, N94, N263, N305, N329, Q467, N479, N514, and N653) from the deamidation sites of the capsid proteins of AAV8 vectors, including those that had an impact on transduction efficiency in the AAV8 vector,^22^^,^^27^ those involved in the immune response in the AAV9 vector,^29^ and/or those conserved in AAV8 were selected for detailed evaluation. Of these, residues N57, N94, and N263, which had a mean deamidation ratio >1% for all products, were used for correlation analysis. The mean deamidation ratios of N57, N94, and N263 of each product obtained by replicate experiments ranged from 8.4% (Prod. 4) to 18.6% (Prod. 8), 1.6% (Prod. 2 and 4) to 2.8% (Prod. 8), and 3.7% (Prod. 2 and 7) to 10.6% (Prod. 8) for the 10 products, respectively (Figures 3A–3C).

### Formulation assessment

**Figure 4:**
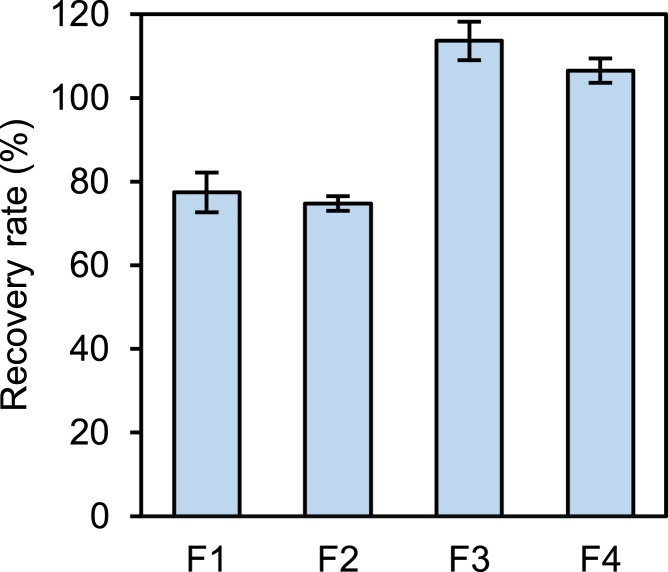
Recovery rates before and after the storage stability study The recovery rates for the samples before and after storage in F1 and F2 decreased significantly, respectively (*p* values = 0.02 and 0.01, respectively). Conversely, the recovery rates of the samples in F3 and F4 did not exhibit significant decreases (*p* values = 0.06 and 0.08, respectively). Error bars indicate standard deviation (SD). The formulation of each buffer is described in the materials and methods section.

Even highly purified AAV vectors, their stability in solution during storage depends on formulation. Formulation assessment focused on recovery rates through the freeze-thaw cycle was performed in the selected conditions. The recovery rates for AAV8 vector before and after storage in formulation (F)1 and F2 decreased significantly, respectively. Conversely, the recovery rates of the samples in F3 and F4 did not decrease significantly (Figure 4).

### Correlation between downstream in-process results and product quality attributes

The correlation between the downstream in-process results and product QAs is depicted in Figure S8. A positive correlation was observed between the yield and stoichiometric ratio of VP3 molecules (Figure S8A). Correspondingly, the negative correlations were observed between the yield and stoichiometric ratio of VP2 molecules, whereas the stoichiometric ratio of VP1 molecules remained unchanged regardless of the yield (Figure S8B). No correlation was found between yield and transduction efficiency (Figure S8C). These results indicate that the higher yield was likely due to increased VP3 production, which, unexpectedly, reduced the stoichiometric ratio of VP2 molecules.

### Correlation between the quality assessment results of AAV8-CMV-EGFP

In this study, an exhaustive pairwise correlation analysis was performed for all 136 combinations of 17 QA parameters measured across 10 products, with the aim of exploratorily identifying potential relationships (Figure S9). This analysis revealed several significant correlations. Among these, the correlations observed between transduction efficiency and either the number of VP molecules or the degree of deamidation were considered to be of particular practical importance. Therefore, the following evaluation focuses on these specific relationships.

#### Relationship between VP-related parameters and transduction efficiency

**Figure 5:**
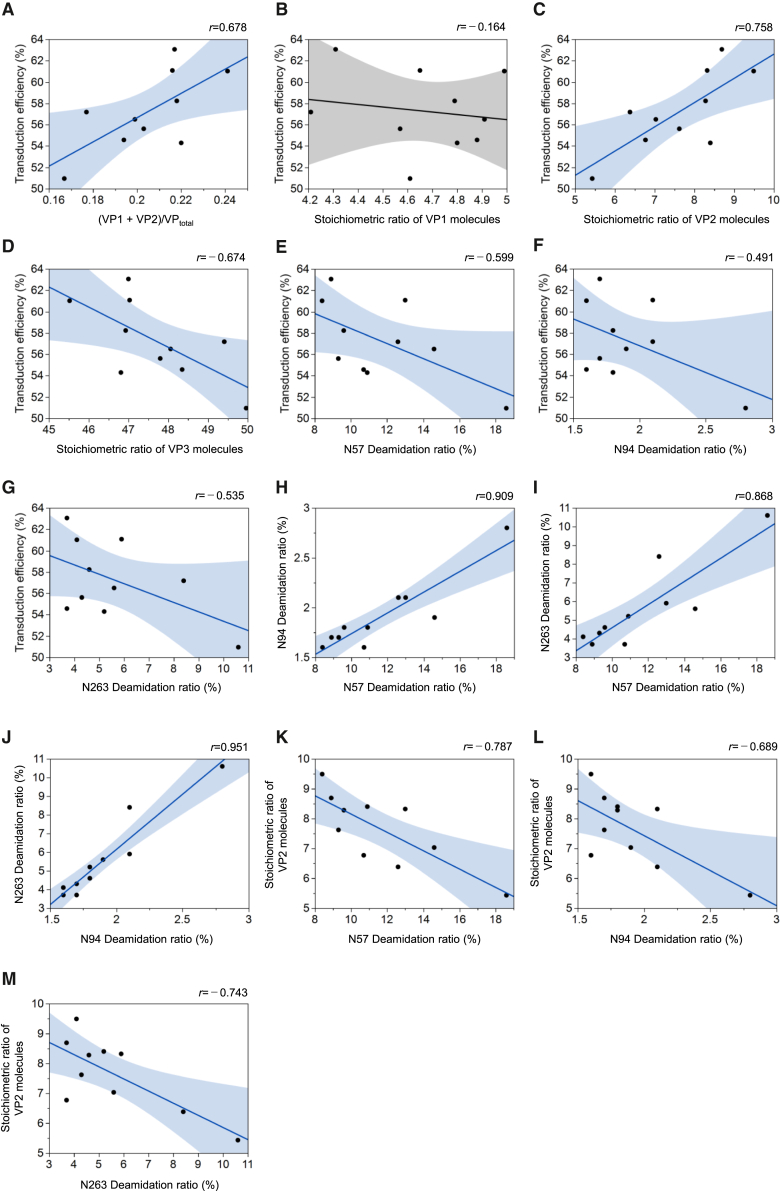
Correlation analysis among the quality attributes (A–M) Scatterplots among several quality attributes (QAs), including the transduction efficiency, the stoichiometric ratios of VPs, the deamidation ratios of Asn residues, with the mean of the QAs for each product plotted. Linear lines indicate regression lines for the 10 data points. Filled areas indicate 95% confidence intervals. For clarity, the confidence intervals are filled in blue when they are significant and gray when they are not. Pearson correlation coefficient was indicated in the upper-right corner of each plot.

Transduction efficiency is one of the most notable QAs considering the primary advantage of using AAV vectors in gene therapy, which is to deliver the therapeutic gene to the target cell to induce the expression of the protein encoded by the GOI. A positive correlation was observed between the mean ratio of the (VP1 + VP2)/VP_total_ and transduction efficiency (Figure 5A). As already described, few variations in VP1 were confirmed, thus no correlation was observed between the stoichiometric ratio of VP1 molecules and transduction efficiency (Figure 5B). Unexpected from the previous study,^7^ a positive correlation was observed between the stoichiometric ratio of VP2 molecules and transduction efficiency. Correspondingly, a negative correlation was observed between the stoichiometric ratio of VP3 molecules and transduction efficiency (Figures 5C and 5D). Therefore, the stoichiometric ratio of VP2 molecules might be considered a positive attribute of transduction efficiency.

#### Relationship between the deamidation ratio and transduction efficiency

Previous study showed that the deamidation of N57, N94, and N263 of the AAV8 capsid protein reduces transduction efficiency.^22^ Consistently, in the present study, a negative correlation was observed between the deamidation ratios of N57, N94, and N263 and transduction efficiency (Figures 5E–5G). Among the AAV1-10 serotypes, N57 and N94 were well conserved in all serotypes. Therefore, the deamidation ratio of N57 and N94 observed in all AAV serotypes might be considered a negative QA of transduction efficiency based on this correlation analysis, which was further examined later in this study.

The deamidation ratios of N57, N94, and N263 were correlated with one another (Figures 5H–5J). Unexpectedly, the stoichiometric ratio of VP2 molecules was negatively correlated with the deamidation ratios of N57, N94, and N263 (Figures 5K–5M). Although the exact reason for this phenomenon is unclear, the steric hindrance caused by additional VP2 molecules may inhibit the deamination of the VP1 unique region, leading to the correlation between the change in the stoichiometric ratio VP2 molecules (i.e., a change in the stoichiometric ratio active VP1 molecules) and the deamidation ratio. Furthermore, these results demonstrated that it was not possible to determine whether changes in the transduction efficiency were due to changes in VP2 stoichiometric ratio or those in the deamidation ratio. It was thought that experiments were needed to investigate the transduction efficiency by changing only either VP2 stoichiometric ratio or deamidation ratio. Since the deamidation ratio of VP is known to change through the short-term storage,^36^ to extract the contribution of deamidation to the transduction efficiency, short-term accelerated stability testing was conducted as described later.

### Distinction of contribution between deamidation ratio and VP2 stoichiometric ratio to transduction efficiency

**Figure 6:**
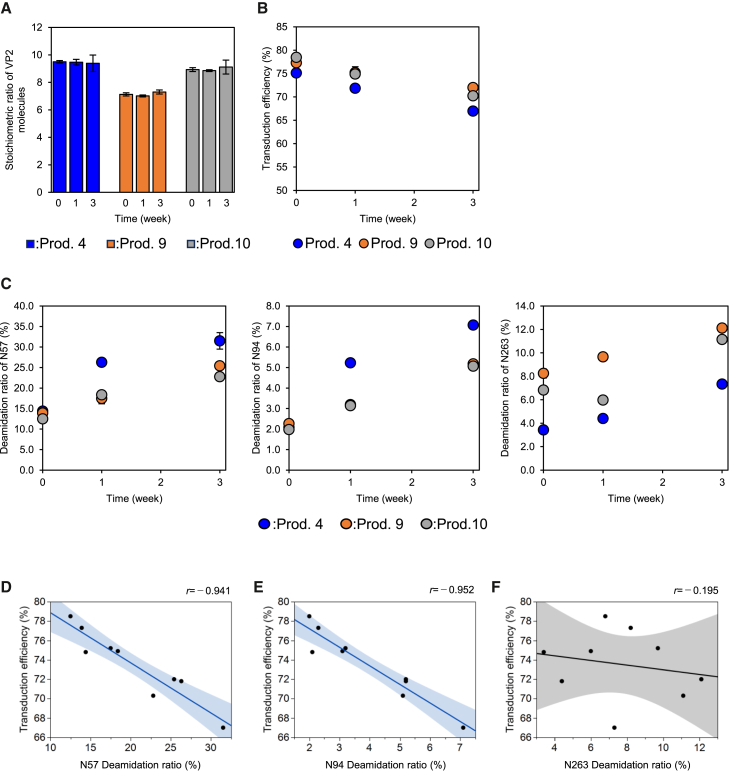
Changes in the deamidation ratio and transduction efficiency associated with short-term accelerated stability testing Changes in the (A) stoichiometric ratio of VP2, (B) transduction efficiency, and (C) deamidation ratios of N57 (left), N94 (middle), and N263 (right). Error bars indicate standard deviation (SD) calculated from experiments performed in triplicate. (D–F) Scatterplot between the deamidation ratio and transduction efficiency. The linear lines indicate the regression lines for the 9 data points. Filled areas indicate 95% confidence intervals. For clarity, the confidence intervals are filled in blue when they are significant and gray when they are not. Pearson correlation coefficient was indicated in the upper-right corner of each plot.

Since the correlation between the deamidation ratios and VP2 stoichiometric ratio was confirmed, the effects of the deamidation of N57, N94, and N263 on the transduction efficiency at a nearly constant VP2 ratio was assessed by short-term accelerated stability testing at 25°C for the selected products (Prod. 4, 9, and 10), where the temperature was previously used to promote the deamidation of Asn residues for the AAV9 vector.^36^ After 3 weeks’ storage, VP2 stoichiometric ratios of all three products did not change (Figure 6A). Meanwhile, the transduction efficiency decreased significantly over the storage period for all three products (Figure 6B). The deamidation ratios of all three Asn residues increased significantly over the storage period for all 3 products (Figure 6C). A negative correlation was found between the deamidation ratios and the transduction efficiency for N57 and N94, but not for N263 (Figures 6D–6F), indicating that deamidation of these Asn residues are negative QA of transduction efficiency.

## Discussion

In this study, through the manufacture and analysis of 10 AAV8-CMV-EGFP products, the variations in the in-process results and the correlations among the QA parameters were evaluated (Figure S1).

The peak area observed for the AC was positively correlated with yield (Figure S1D). In other words, yield can be estimated in the early stage of the downstream process. Similarly, yield can be estimated from the peak area observed for the DG-UC (Figure S1E). Through the downstream process, including AC and DG-UC, the full-empty ratio can be increased. The enrichment of full particles can also be achieved by ion-exchange chromatography, as reported in recent studies,^37^^,^^38^^,^^39^ in which yield can be estimated using the peak area.

The yield was significantly negatively correlated with the stoichiometric ratio of VP2 molecules and significantly positively correlated with the stoichiometric ratio of VP3 molecules (Figures S8A and S8B). The VP2 and VP3 proteins are translated from the same mRNA species.^40^^,^^41^ VP3 proteins, which contains the canonical initiation codon AUG, are translated more than VP2 proteins, which have the noncanonical initiation codon ACG.^42^ The positive correlation between the yield and stoichiometric ratio VP3 molecules may be explained as follows: the more VP proteins that are translated, the greater the number of capsid particles formed in the nucleus and the population of capsid particles with more VP3 molecules, i.e., the population with fewer VP2 molecules. Because yield has a weak negative trend with transduction efficiency in this study, the enhanced production of AAV vectors, including therapeutic DNA, did not result in the acquisition of therapeutically effective vectors. One possible strategy for achieving high yield and transduction activity is to employ a mutant AAV vector that introduces a mutation at the VP3 translation initiation site, as demonstrated in a recent study.^20^ Specifically, the AAV2-M203V and AAV2-M211V vectors exhibited significantly increased transduction efficiency compared with the wild-type AAV2 vector without yield reduction.^20^

To administer manufactured AAV vectors to patients, the formulation conditions must be optimized along with the AAV vector product by altering the properties of the capsid protein, as described previously. Phosphate buffer is a common formulation in AAV vector-based therapeutics; however, freezing and thawing in phosphate buffer decreases the genomic titer.^43^ Because several freeze-thaw cycles are conducted during the fill-and-finish and pre-administration processes,^44^ a formulation condition that does not affect QAs directly related to the activity of the AAV vector, such as genome titers and gene transfer efficiency, must be identified, even if the vector undergoes freeze-thaw cycling. In our experiment on the change in genomic titer following freeze-thaw cycles (Figure 4), we found that freeze-thaw processes performed in phosphate buffer significantly decreased the genomic titer but not in Tris buffer containing sucrose or arginine, which we used based on previous studies.^45^^,^^46^ In addition to genomic titer, particle size, size distribution, and transduction efficiency, which have been investigated in previous studies, assessing the deamidation ratio, which was negatively correlated with transduction efficiency in this study, provides a new research perspective when the effect of freeze-thaw cycles on the QAs of AAV vectors is evaluated.

The stoichiometric ratio of VP2 molecules was positively correlated with the transduction efficiency (Figure 5C). A VP2 molecule contains two basic amino acid clusters in the N-terminal region that do not overlap with the amino acid sequence of the VP3 molecules.^17^ The basic amino acid cluster on the C-terminal side of the VP1/VP2 common region is essential as a nuclear localization signal (NLS) in nuclear transfer in AAV2.^8^ As a result, products with a large stoichiometric ratio of VP2 molecules have high transduction efficiency. A previous study demonstrated that VP1, which has both a phospholipase A2 domain for endosomal escape and a NLS for nuclear transfer, and VP3, which is involved in capsid integrity and receptor binding, are essential in the transduction of the GOI to target cells (hereinafter referred to as gene transduction) in AAV vectors.^7^ In contrast, VP2, which only has NLS, is nonessential for gene transduction.^7^ Therefore, combining previous findings with the present results, although not essential for gene transduction, the stoichiometric ratio of VP2 in the capsid is a QA that influences transduction efficiency.

Although it has been shown that the deamidation of specific Asn residues of AAV8 vector may have a negative impact on transduction efficiency,^22^ the relationship between the residue number and the deamidation ratio of each Asn residue and transduction efficiency is not fully understood. This is partly because in the mutagenesis experiments that were used, it was not possible to rule out the possibility that alters occurring in sites other than the mutation site could affect the transduction efficiency. In this study, through the quantitative evaluation using mass spectrometry, N57, N94, and N263 were confirmed as deamidation hotspots, which can be explained in terms of the accessibility of these Asn residues. Recent hydrogen/deuterium exchange mass spectrometry has shown that N57 and N94 are located in a region highly accessible to water molecules.^47^^,^^48^ N263 is exposed on the surface, as evident from higher-order structure analysis.^49^ The correlation was observed among the deamidation ratios of N57, N94, and N263 (Figures 5H–5J), demonstrating that these three residues concurrently undergo deamidation. The trend of the transduction efficiency decrease was confirmed as the deamidation ratios of N57 and N94 in VP1 unique region and that of N263 in VP3 region increase. Meanwhile, Asn residue with a more remarkable influence on the transduction efficiency of the AAV vector was not confirmed in this study, mainly because of less deamidation event for other Asn residues during the manufacturing. Further assessment of the deamidation effect for these three Asn residues on the transduction efficiency by the acceleration testing identified the significant contribution of the deamidation to the reduced transduction efficiency for N57 and N94 in VP1 unique region but not for N263 in VP3 region. Thus, at least deamidation ratios of N57 and N94 should be monitored as pCQA of AAV vectors.

Comparison between Prod. 4 and 6 has shown that when the deamidation ratios of N57 and N94 are the same level (Student’s t test *p* value ≥ 0.05) whereas the higher VP2 stoichiometric ratio, the higher transduction efficiency. A similar phenomenon was observed in the comparison of Prod. 9 and 10 (Table S2). These comparisons support one of the conclusions that VP2 stoichiometric ratio is a positive factor for transduction efficiency. Additionally, as confirmed in the short-term accelerated stability testing, even when the VP2 stoichiometric ratio is the same level (Student’s t test *p* value ≥ 0.05) but the higher deamidation ratio, the lower transduction efficiency. These results provided the fact that both VP2 stoichiometric ratio and deamidation at N57, N94, and N263 are QAs that correlate to transduction efficiency independently.

**Figure 7:**
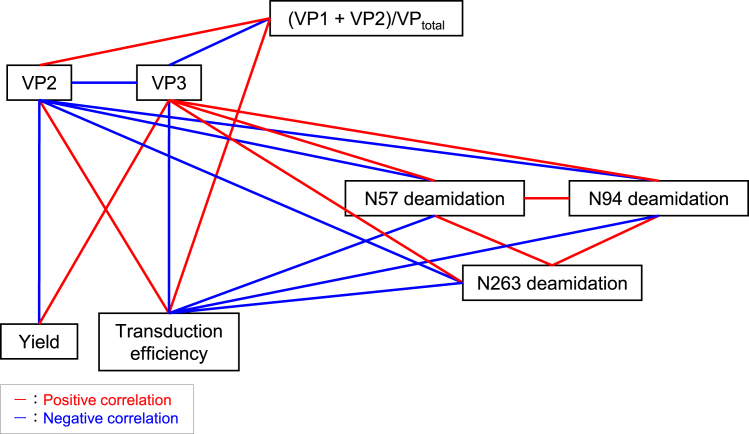
Summary of the correlations among the quality attributes The red and blue lines indicate significantly positive and negative correlations, respectively.

As a result of 14 QA evaluations of the 10 AAV8-CMV-EGFP products, QAs that showed significant correlations among them are shown in Figure 7. Most importantly, among these QAs, the stoichiometric ratio of VP2 had a significant positive correlation and deamidation in VP1 had a negative trend/correlation with the transduction efficiency.

In summary, our study clearly demonstrated that the stoichiometric ratio of VP2 molecules and deamidation ratios of N57 and N94 could be pCQAs of AAV vector products in terms of gene transduction.

For the future aspect, the QA parameters which vary in batch-to-batch and influence the quality of AAV vector identified in this study could be useful for the process development of AAV vector manufacturing, for example, for the establishment of a design space using DoE (design of experiments) approach in the process development of AAV vector manufacturing. In DoE, it is essential to know which QA can be a CQA for the establishment of a design space for CPPs (critical process parameters) (e.g., temperature, pH, stirring speed, and material concentration). In this study, we were able to conclude that at least the VP2 stoichiometric ratio and the deamidation ratios of N57 and N94 will be able to be used in the identification and the determination of the range of CPPs.

## Materials and methods

### Chemical reagents

Poloxamer 188 powder (BASF, Ludwigshafen, Germany) was used to prepare all Poloxamer 188 solutions at varying concentrations as required for each experiment. Unless otherwise specified in the individual experimental sections, reagents were primarily obtained from Fujifilm Wako Pure Chemical (Osaka, Japan) or Nacalai Tesque (Kyoto, Japan) and used without further purification.

### Vector manufacturing and downstream process

All AAV vectors were generated by triple transfection. Specifically, AAV vectors were manufactured under the same conditions, except for the culture volume and container type. Batch A was prepared as a 0.2-L culture in a 1-L flask, after which cell lysis and purification were performed. This cycle was repeated four times. Batch B was prepared as a 1-L culture in a 2-L flask, after which cell lysis and purification were performed. This cycle was repeated three times. Batch C was prepared as a 1-L culture in a 2-L bioreactor, after which cell lysis and purification were performed. This cycle was repeated three times. To exclude the influence of variations in purification, in the case of the 1-L culture, the lysed solution was divided into 5 equal volumes; thus, a uniform volume was applied for purification regardless of the culture volume. In total, three batches of the 10 AAV vector products were prepared.

In detail, transgene plasmids (CMV-EGFP), pAAV-Rep&Cap (serotype 8) and pAd helper were cotransfected into suspended VPC (viral production cells) 2.0 cells (Thermo Fisher Scientific, Waltham, MA, USA) at 2.0 × 10^6^ cells/mL and cultured in a flask or bioreactor at a plasmid ratio of 1:1:1. FectoVIR-AAV (Polyplus, Illkirch, France) was used as the transfection reagent. At 4 days after transfection, the medium and cell lysates were harvested, filtered, and purified by AC using AAVX prepacked columns (Thermo Fisher Scientific). The purified AAV samples were then subjected to CsCl ultracentrifugation to separate the full and empty particles. The purified samples containing 3 or 3.5 M CsCl were centrifuged at 18,000–25,000 rpm in an Optima XE-90 centrifuge using a Beckman SW41Ti rotor (Beckman Coulter, Brea, CA, USA) at 16°C for 24–42 h. The full particle fractions were collected using an online monitoring apparatus and dialyzed in 200 mM NaCl, 0.001 w/v% Poloxamer 188, and 1× PBS buffer using Slide-A-Lyzer G2 or Slide-A-Lyzer G3 dialysis cassettes (Thermo Fisher Scientific), followed by filtration using a 0.22-μm Millex-GV syringe filter unit (Millipore, Burlington, MA, USA).

Monitoring of the downstream process was performed for the AC peak area, A260/A280 after AC, mean DG-UC peak area, A260/A280 after DG-UC, yield, and A260/A280 of the product. The in-process results for the downstream processes were evaluated for their possible correlation with one another and with the QAs of the AAV8-CMV-EGFP products.

### Genomic titer determination

AAV vector-containing samples were diluted in dilution buffer, which consisted of DNase I Buffer, DNase I (Takara Bio, Shiga, Japan), and 0.05 w/v% Poloxamer 188. The mixtures were incubated at 37°C for 30 min to digest DNA contaminants outside the AAV vector capsid. DNase I was inactivated by adding EDTA (pH 8.0) (NIPPON GENE, Tokyo, Japan) to a final concentration of 50 mM. The samples were then heated at 95°C for 10 min to denature the AAV vector capsid. The pretreated samples were diluted with 0.001 w/v% Poloxamer 188 in Tris-EDTA (TE) buffer (Invitrogen, Waltham, MA, USA) to the required concentration. Digital polymerase chain reaction (dPCR) was performed as a duplex assay using specific forward and reverse primers (900 nM) and a specific probe (250 nM) that targeted the ITR using a fluorescein amidite (FAM) fluorophore (Hokkaido System Science, Hokkaido, Japan). Reaction mixes comprised primers, probes, nuclease-free water, and QuantStudio Absolute Q DNA Master Mix (Thermo Fisher Scientific). dPCR was performed using a QuantStudio Absolute Q Digital PCR System and 16-well microfluidic array plates (Thermo Fisher Scientific). The dPCR sequence consisted of enzyme activation at 96°C for 10 min, followed by 40 cycles of 94°C for 5 s and 54°C for 30 s. The following primers and probes were used: ITR forward primer 5′-GGAACCCCTAGTGATGGAGTT-3′; ITR reverse primer 5′-CGGCCTCAGTGAGCGA-3′; and ITR probe 5′-[FAM]-CACTCCCTCTCTGCGCGCTCG [BHQ1]-3′.

### Cryo-EM analysis

Holey carbon grids (Quantifoil Ultra-Cu R1.2/1.3 300 mesh) coated with carbon film were used to mount the frozen specimens. The grids were glow-discharged for 30 s using a VES-10 multicoating unit (Vacuum Device, Ibaraki, Japan) before use. Sample solution (3 μL) was applied to the grids for rapid freezing, which was performed using a Vitrobot Mark IV (Thermo Fisher Scientific). All samples were examined using a Tundra Cryo-TEM (Thermo Fisher Scientific) at liquid nitrogen temperature at an accelerating voltage of 100 kV. Images were recorded in the automated acquisition mode at a nominal magnification of 110,000× under 4-μm focus conditions.

Particle images of the AAV vector were cropped from the acquired micrographs, as described previously.^31^ Whole particle images were classified into full and empty particles using the CNN classifier. The trained CNN on the AAV9 image dataset, as described previously,^31^ was adopted. The trained CNN was implemented using MATLAB R2019b (version 9.7.0.1216025, The MathWorks, Natick, NJ, USA). The full-empty ratios were calculated based on the number of full and empty particle images identified.

### UV spectroscopy

Ultraviolet-visible (UV-vis) measurements were performed using a NanoDrop One (Thermo Scientific, Waltham, MA, USA), BioMate 160 UV–Vis (Thermo Scientific), or UV-1900 (Shimadzu, Kyoto, Japan) spectrophotometer. The mean absorbance at 260 nm (A260) and 280 nm (A280), A260/A280 ratio, and SD were calculated from triplicate experiments.

### Dynamic light scattering

The Z-average diameter and D90 of the AAV vector samples were determined using a Zetasizer Ultra (Malvern Panalytical, Malvern, UK) at 25°C. A 30-μL sample was loaded into the cuvette. The mean of each parameter and SD were calculated from triplicate experiments.

### Capillary gel electrophoresis

#### VP assessment

A 1-μg AAV vector sample was mixed with 14.4 μL of 10% sodium dodecyl sulfate (SDS; NIPPON GENE) and 4.83 μL of 2-mercaptoethanol (Nacalai Tesque, Kyoto, Japan) in 0.80 mL and incubated at 70°C for 3 min. The buffer was replaced twice with a 70% matrix exchange solution consisting of 3.5% of 2-mercaptoethanol and 0.033% SDS using Amicon (Merck Millipore, Burlington, MA, USA). The collected samples were incubated at 70°C for 3 min. Then, a 1-μL mixture of 20-fold diluted 10 KD Internal Standard (SCIEX, Framingham, MA, USA) and Milli-Q water was added to yield a total volume of 70 μL. A PA800 Plus Pharmaceutical Analysis CE system (SCIEX) equipped with a photodiode array detector (214 nm) and 32 Karat software (version 10.3, Build 20) was used for all experiments. Bare-fused silica capillaries (50 μm I.D., 30 cm total length, and 20 cm effective length; SCIEX) were used for separation. Data acquisition and analysis were performed using 32 Karat Software (version 10.3 Build 20). After correcting the corrected area of each VP peak using the extinction coefficient at 214 nm, the stoichiometric ratio of VP molecules in the 60-mer was calculated. The mean VP stoichiometric ratio; the stoichiometric ratio of VP1, VP2, and VP3 molecules; and the SD were calculated from triplicate experiments.

#### Nucleic acid assessment

Nucleic acids in each sample were extracted using Proteinase K (QIAGEN, Venlo, Netherlands). The treated samples were purified using a QIAquick PCR Purification Kit (QIAGEN). The extracted DNA was analyzed using a PA800 Plus system (SCIEX) with a laser-induced fluorescence detector. Fluorescence was detected by excitation at 488 nm and emission at 520 nm. Separations were performed using a bare-fused silica capillary with an inner diameter of 50 μm and length of 30 cm (SCIEX). Separations were performed using a running buffer containing Nucleic Acid Extended Range Gel (SCIEX) and SYBR Green Ⅱ RNA Gel Stain (SCIEX). A single-stranded DNA 7-K Ladder (PerkinElmer, Shelton, CT, USA) was used as the size standard. The electropherograms were analyzed using 32 Karat Software version 10.3.

### BS-AUC analysis

BS-AUC experiments and analyses were conducted as described previously.^50^ Briefly, 15 μL of the buffer or sample was loaded into a reference or sample reservoir well with a 12-mm band-forming centerpiece (Spin Analytical, South Berwick, ME, USA) equipped with sapphire windows. Then, 250 or 240 μL of PBS dissolved in 98 atom% H_2_^18^O (Rotem, Dimona, Israel) containing 0.001 w/v% Poloxamer 188 was loaded into the reference or sample sector, respectively. Data were collected at 20°C using an Optima AUC (Beckman Coulter) at 20,000 rpm with a UV detection system and a detection wavelength of 230 nm. Data were collected at a radial increment of 10 μm every 150 s.

The sedimentation data were analyzed using the analytical zone centrifugation c(s) model of the SEDFIT program (version 16.2b)^51^ with a fitted lamella width, frictional ratio, meniscus, time-invariant noise, and radial-invariant noise and a regularization level of 0.68. The s-value range of 0–175 S were evaluated at a resolution of 350 nm. The buffer density and viscosity of the solvent loaded into the sectors were calculated using the SEDNTERP program.^52^ The apparent sedimentation coefficient of the full particle was converted to the sedimentation coefficient in water at 20°C, *s*_20,w_ using the buffer density, buffer viscosity, and partial specific volume of the full particle, the latter of which was calculated as described previously.^53^ The particle concentration was calculated by dividing the full and empty peak areas using the molar extinction coefficient at the set detection wavelength. The full-empty ratio was calculated from the particle concentration ratio (=F/[E + F]). The *s*_20,w_, full-empty ratio, and SD of each parameter were calculated from triplicate experiments.

The *s*_20,w_ values of full particles were used to calculate the nucleic acid lengths of the particles. The following equation was derived from the BS-AUC experiments in PBS/H_2_^18^O containing 0.001 w/v% Poloxamer 188 using AAV vectors with different nucleic acid lengths^54^:

(Equation 1) $$DNA\;length\;\left(base\right)=104.81\times s_{20,w}-7194$$

### Flow cytometry

HeLaRC32 cells were seeded at 5 × 10^4^ cells/well in 24-well plates in 0.5 mL of 10% fetal bovine serum (HYCLONE, Marlborough, MA, USA) containing DMEM (Sigma-Aldrich, Burlington, MA, USA). We infected cells with AAV vectors at an MOI of 5 × 10^4^ in triplicate. Cells were incubated at 37°C for 2 days and then harvested. The percentage of viable cells expressing EGFP was determined using the CytoFLEX flow cytometry system (Beckman Coulter).

### Enzyme-linked immunosorbent assay for HCP quantitation

The HCP content was quantified using an HEK 293 HCP enzyme-linked immunosorbent assay kit, 3G (Cygnus Technologies, NC, USA) according to the manufacturer’s instructions. A four-parameter logistic fit was used to obtain a standard curve to quantify the HEK293-derived HCP in the samples. The HCP content was normalized to the protein content of each product. The mean HCP content and SD were calculated from experiments.

### qPCR analysis of host cell DNA quantitation

DNA was extracted from AAV vector-containing samples using a QIAquick PCR Purification Kit (QIAGEN), and the extracted DNA was quantified using a resDNASEQ Human Residual DNA Quantitation Kit (Thermo Fisher Scientific). Both procedures were performed in accordance with the manufacturers’ instructions.

### Peptide mapping for deamidation ratio analysis

A 7.5-μg AAV vector sample was incubated with 50 mM ammonium bicarbonate (Fujifilm Wako Pure Chemical, Osaka, Japan), 6 M guanidine hydrochloride (Fujifilm Wako Pure Chemical), 5 mM Tris(2-carboxyethyl)phosphine hydrochloride (Fujifilm Wako Pure Chemical), and 5 mM iodoacetamide (Fujifilm Wako Pure Chemical) at 20°C for 1 h in the dark. The denatured AAV was diluted 10-fold with water containing 0.1 v/v% formic acid (FA; Kanto Chemical, Tokyo, Japan) and applied to a MonoSpin S-type C18 column (GL Science, Tokyo, Japan). The applied sample was washed with distilled water containing 0.1 v/v% FA and water/acetonitrile (2/8) containing 0.1 v/v% FA and eluted with acetonitrile containing 0.1 v/v% FA. The eluted AAV was lyophilized and redissolved in 30 μL of 50-mM ammonium acetate at pH 6.5. Rapyzime trypsin (Waters, Milford, MA, USA) and lysyl endopeptidaseR (Lys-C; Fujifilm Wako Pure Chemical) were mixed at a trypsin-to-Lys-C-to-protein ratio of 0.66:0.33:30 and incubated at 37°C for 4 h. Digestion was stopped by adding 3 μL of 1 v/v% trifluoroacetic acid (Tokyo Chemical Industries, Tokyo, Japan). An Ultimate 3000 LC pump (Thermo Fisher Scientific) coupled to a Q Exactive HF-X mass spectrometer (Thermo Fisher Scientific) was used for liquid chromatography-tandem mass spectrometry (LC-MS/MS) measurements. The peptides were collected in an Acclaim Pepmap100 C18 column (0.3 × 5 mm, 5 μm particle size; Thermo Fisher Scientific) and separated using an ACQUITY UPLC Peptide CSH C18 Column (130 Å, 1.7 μm; 1.0 × 150 mm; 1.7 μm particle size). Mobile phase A consisted of water containing 0.1 v/v% FA, and mobile phase B consisted of acetonitrile containing 0.1 v/v% FA. The LC condition for separation was a gradient of 5%–35% for mobile phase B for 45 min at 50 μL/min. A full MS scan was performed at an ion transfer tube temperature of 250°C, resolution of 120,000, mass range (m/z) of 300–2,000, and funnel radio frequency (RF) level of 40. A subsequent data-dependent MS/MS scan was performed with a higher energy collisional dissociation of 27% and a resolution of 30,000. Peptide identification was performed using a Byos v.5.6.68 (Protein Metrics, Cupertino, CA, USA) and BioPharma Finder v.3.2 (Thermo Fisher Scientific). Quantification was performed using Quan Browser in Xcalibur v.4.3.73 11 (Thermo Fisher Scientific).

### Storage stability study

Comparisons of formulations suitable for AAV8 were performed using four different buffers (F1, F2, F3, and F4). F1 buffer (pH 7.2) contained 2.97 mM sodium phosphate dibasic anhydrous, 1.06 mM potassium phosphate monobasic, 355 mM NaCl, and 0.001 w/v% Poloxamer 188. F2 buffer (pH 7.6) contained 10 mM sodium phosphate dibasic anhydrous, 1.47 mM potassium phosphate monobasic, 100 mM NaCl, 2.7 mM KCl, 0.001 w/v% Poloxamer 188, and 4 w/v% (117 mM) sucrose. F3 buffer (pH 7.4) contained 20 mM Tris-HCl, 100 mM NaCl, 2 mM MgCl_2_, 0.001 w/v% Poloxamer 188, and 2 w/v% (58.4 mM) sucrose. F4 buffer (pH 7.4) contained 20 mM Tris-HCl, 100 mM NaCl, 2 mM MgCl_2_, 0.001 w/v% Poloxamer 188, and 90 mM Arg. F2 and F3 were partially modified as described previously.^45^^,^^46^ After each buffer was dialyzed and filtered by centrifugation, the sample concentrations were adjusted to approximately 2 × 10^12^ vg/mL. Half of the adjusted samples were used for the storage study. The storage study was performed as described by Bee et al. in “Compounded (‘Daisy-Chain’) Freeze-Thaw and Time-out-of-intended Storage Studies.”^46^ Briefly, samples were exposed to five freeze-thaw (F/T) cycles between approximately −80°C and room temperature, maintained at 2°C–8°C for 120 h (5 days), and then stored in a box (protected from light) at room temperature for 78 h (3.25 days). The samples were frozen immediately after storage until dPCR was performed. Titers before and after storage were measured using dPCR. The recovery rates, which represent the ratio of the viral load (vg) after storage relative to that before storage, were calculated.

In addition, the relationships among the deamidation ratio of N57, N94, or N263 and the transduction efficiency were investigated by short-term accelerated stability testing. Prod. 4, 9, and 10, formulated and dialyzed in 200 mM NaCl, 0.001 w/v% Poloxamer 188, 1× PBS buffer were stored at 25°C for 1 and 3 weeks. The deamidation ratios of N57, N94, and N263 and the transduction efficiency were subsequently investigated. The experimental methods for each evaluation followed the procedures described in this manuscript.

### Statistical analysis and illustration

Statistical analyses were performed using JMP Pro (version 16.0.0) (SAS Institute, Cary, NC, USA).

For the correlation analysis, the presence or absence of correlation was determined based on the Pearson correlation coefficient. The focus was on correlations that showed a moderate or higher correlation. The criterion is based on literature.^55^ If the absolute value of the Pearson correlation coefficient was 0.4 or more, it was determined that there was a correlation between the two QA parameters. Scatterplots were generated using JMP Pro (version 16.0.0) (SAS Institute).

The Student’s t test was employed to assess the presence of statistically significant variations in a QA between the two products. When the *p* value was less than 0.05, the null hypothesis that no difference exists among the mean values of a QA parameter of two products was rejected in favor of the alternative hypothesis that a significant difference exists in the mean values of them.

## Data and code availability

The data supporting the findings of this study are available from the corresponding author upon reasonable request.

## Acknowledgments

This study was supported by a grant-in-aid from the “Research and development of core technologies for gene and cell therapy” supported by the 10.13039/100009619Japan Agency for Medical Research and Development (grant numbers JP22ae0201001, JP22ae0201002, JP24bk0304007 and JP24se0123004). The authors thank Masanori Noda (U-Medico Inc.), Kentaro Ishii (U-Medico Inc.), Kenjiroo Matsumoto (U-Medico Inc.), and Takayuki Onishi (Osaka University) for their helpful discussions and technical supports.

## Author contributions

S.U. conceived the study. M.F., Y.T., A.M., T.K., and T.U. performed the vector production. Y.T., A.M., and H.N. performed the cryo-EM experiment and data analyses. T.M., K.H., and M.O. performed the UV and AUC experiments and data analyses. T.M. performed the DLS experiments and data analyses. K.B. and R.S. performed the CE experiments and data analyses. Y.Y., M.M., G.K.-B., Y.N., and D.H. performed the peptide mapping experiments and data analyses. T.M. and S.U. summarized the results of each experiment and wrote the manuscript. All authors have edited the manuscript.

## Declaration of interests

T.M., M.F., and M.O. reports a relationship with U-Medico Inc. that includes employment; S.U. reports a relationship with U-Medico Inc. that includes being a founder and CSO; M.M. reports a relationship with Osaka Consolidated Laboratory that includes employment; Y.N. and D.H. reports a relationship with Thermo Fisher Scientific that includes employment.

## Supplemental information

Document S1. Figures S1–S9 and Tables S1 and S2

Document S2. Article plus supplemental information
