# Supplementary material for: Variation of VP2 stoichiometry and deamidation of VP1 during production and their impacts on the transduction efficiency of AAV vectors
Source: Mol Ther Methods Clin Dev. 2025 Sep 1;33(4):101581. doi: 10.1016/j.omtm.2025.101581 (PMC12475848; doi:10.1016/j.omtm.2025.101581)
Supplement: Document S1. Figures S1–S9 and Tables S1 and S2 [file mmc1.pdf]

OMTM, Volume *33*

Supplemental information

Variation of VP2 stoichiometry and deamidation of VP1 during production and their impacts on the transduction efficiency of AAV vectors

Takahiro Maruno, Mitsuko Fukuhara, Yasuo Tsunaka, Aoba Matsushita, Kiichi Hirohata, Karin Bandoh, Megumi Onaka, Risa Shibuya, Yuki Yamaguchi, Haruka Nishiumi, Yoshiki Nagashima, Daisuke Higo, Toshie Kuwahara, Tomoko Ueno, Masaharu Maeda, Guirong Kanai-Bai, Noriko Yamano-Adachi, Tetsuo Torisu, Takeshi Omasa, and Susumu Uchiyama

SUPPLEMENTAL FIGURES

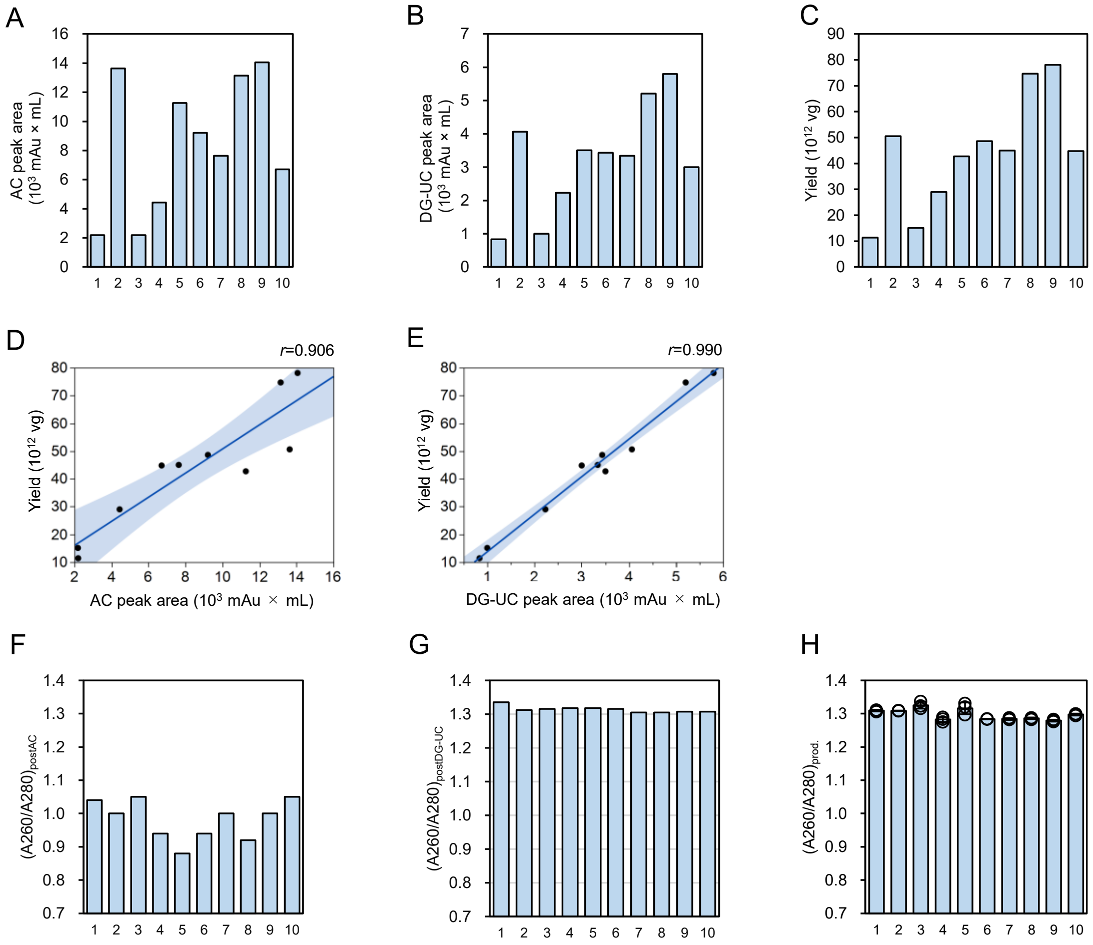

Figure S1. In-process results in downstream and their product-to-product variation of 10 AAV8-CMV-EGFP products.

(A–C) Bar graphs showing the parameters of the 10 products. (A) AC peak area, (B) DG-UC peak area, and (C) Yield. (D) Scatter plot between the AC peak area and yield. (E) Scatter plot between the DG peak area and yield. (F–H) Bar graphs showing the parameters of the 10 products for each in-process result. (F) $(A260/A280)_{postAC}$, (B) $(A260/A280)_{postDG\text{-}UC}$, and (C) $(A260/A280)_{product.}$. The numbers shown in the horizontal lines in A–C and F–H represent the product number. For the correlation plots, the linear lines indicate the regression lines for the 10 data points. Pearson correlation coefficient was indicated in the upper-right corner of each correlation plot. Filled areas denote the 95% confidence intervals.

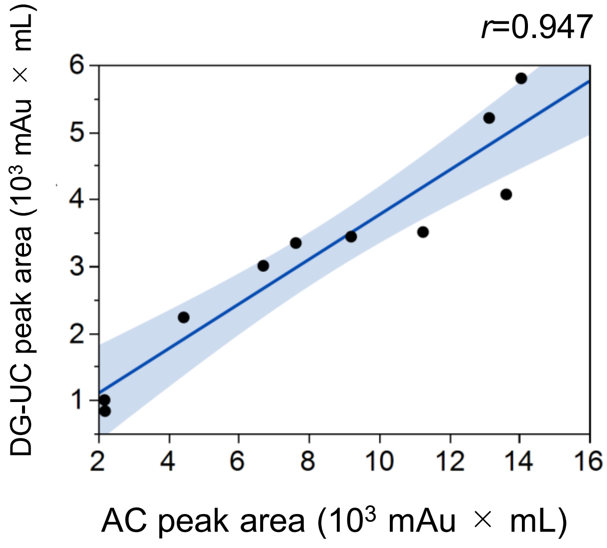

Figure S2. Correlation analysis between peak areas of AC and DG-UC.

Scatter plot between the AC and DG-UC peak areas. The average of the in-process results is plotted. Linear lines indicate regression lines for the ten data points. Filled areas indicate the 95% confidence intervals. Pearson correlation coefficient was indicated in the upper-right corner of each correlation plot.

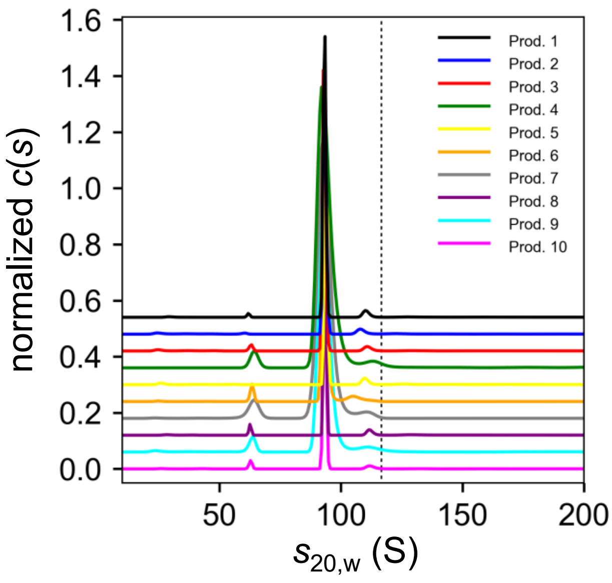

Figure S3. Overlay of the representative sedimentation coefficient distributions.

Dotted line represents the $s_{20,w}$, calculated using Eq. 1 in the manuscript, of the particle with two intact ssDNA molecules. The figure was generated using the program GUSSI (version 1.1.0).[1]

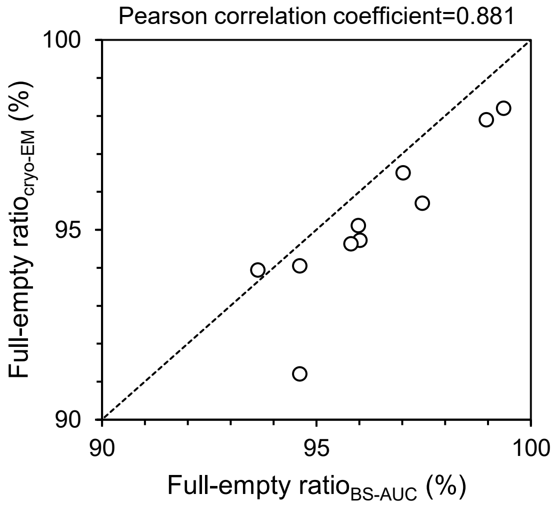

Figure S4. Correlation of full-empty ratios between cryo-EM and BS-AUC.

The dashed line represents the y=x line.

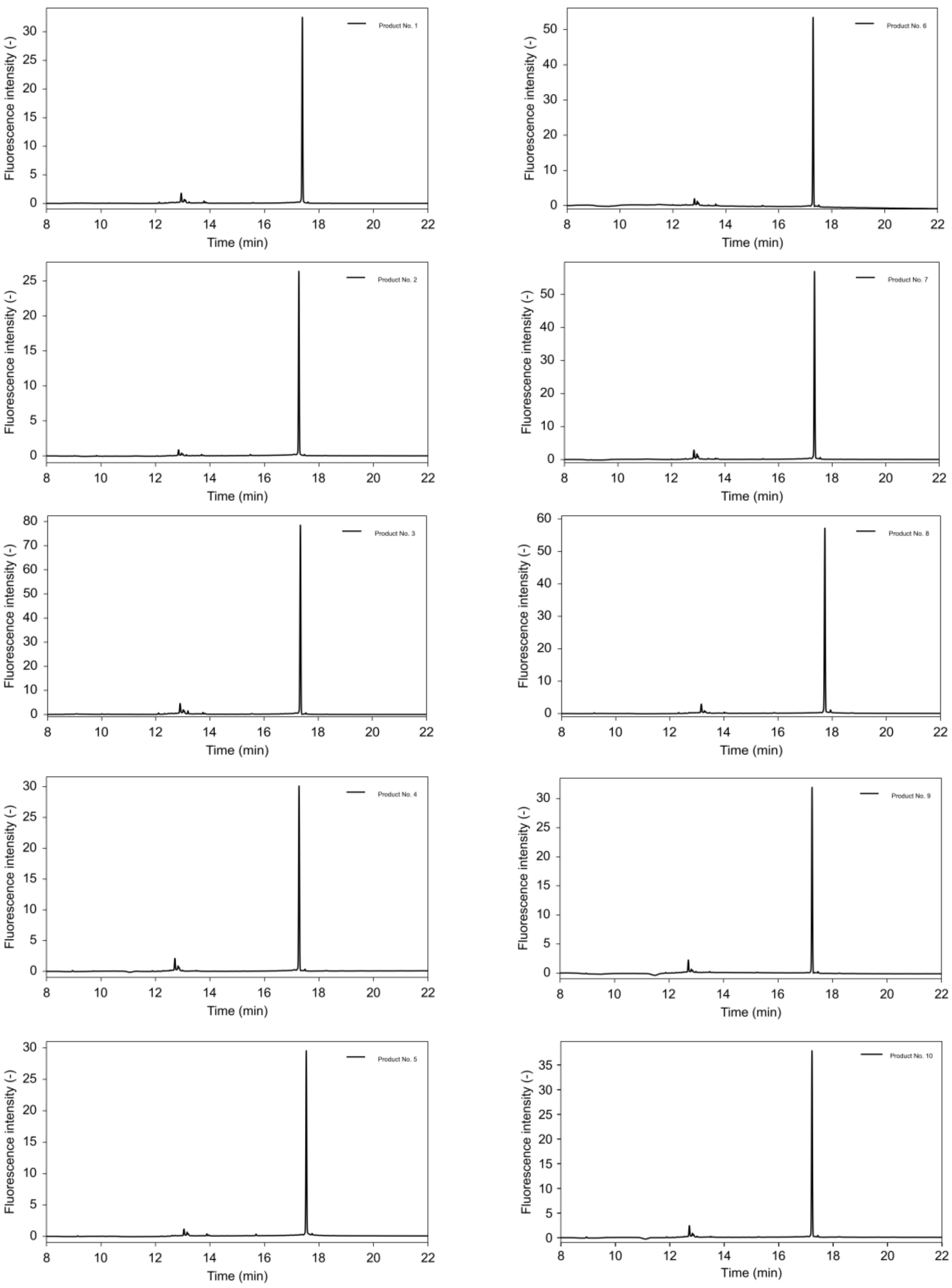

Figure S5. Representative electropherogram of CGE for nucleic acid assessment.

The product number was shown in the upper-right corner of each figure.

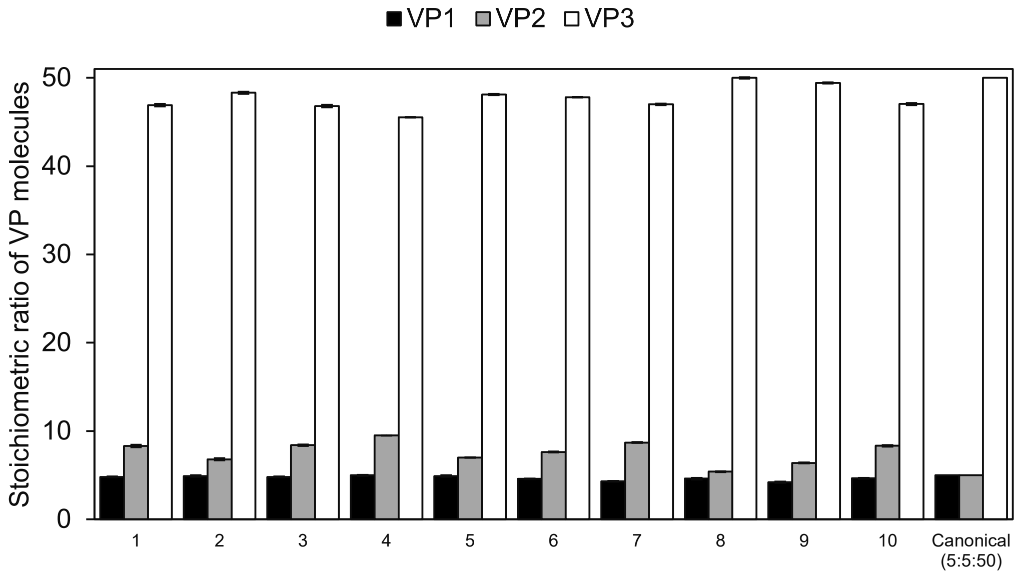

Figure S6. Stoichiometric ratio of VP molecules of 10 products.

The stoichiometric ratio of VP1, VP2, and VP3 molecules were shown as black, gray, and white bar, respectively. The right-side bar graph represents the canonical stoichiometric ratio of VP molecules. Error bars indicate standard deviation calculated from experiments performed in triplicate.

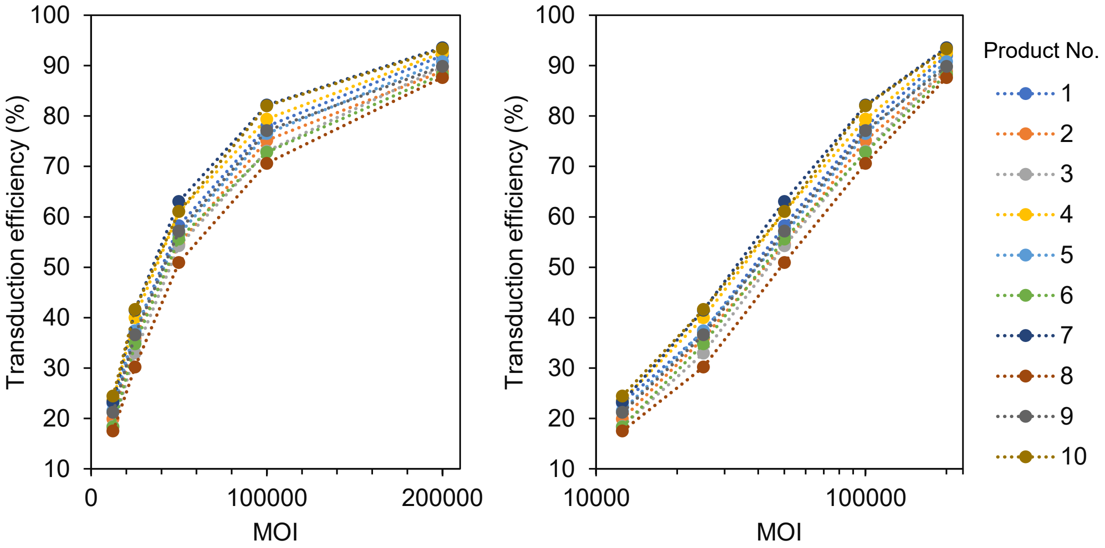

Figure S7. MOI dependence of the transduction efficiency.

(Left) MOI dependence of the transduction efficiency. (Right) Horizontal axis of the left figure converted to log scale.

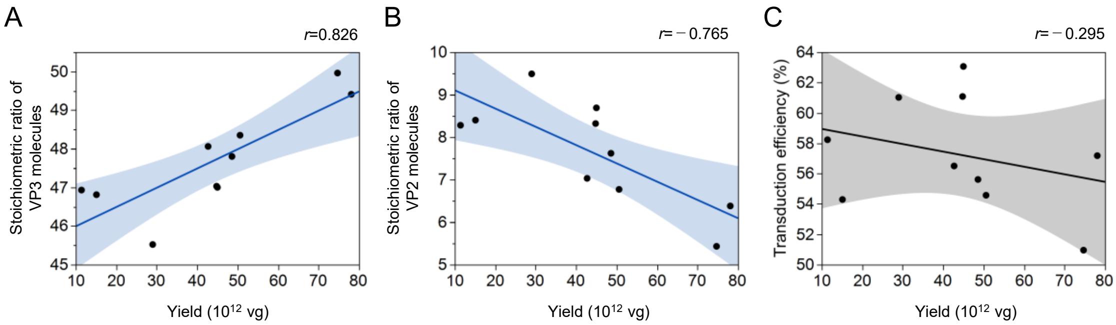

Figure S8. Correlation analysis among the quality attributes (QAs).

(A–C) Scatter plots among QAs. The average of the QAs for each product is plotted. Linear lines indicate regression lines for the ten data points. Filled areas indicate the 95% confidence intervals. For clarity, the confidence intervals are filled in blue when they are significant and gray when they are not. Pearson correlation coefficient was indicated in the upper-right corner of each correlation plot.

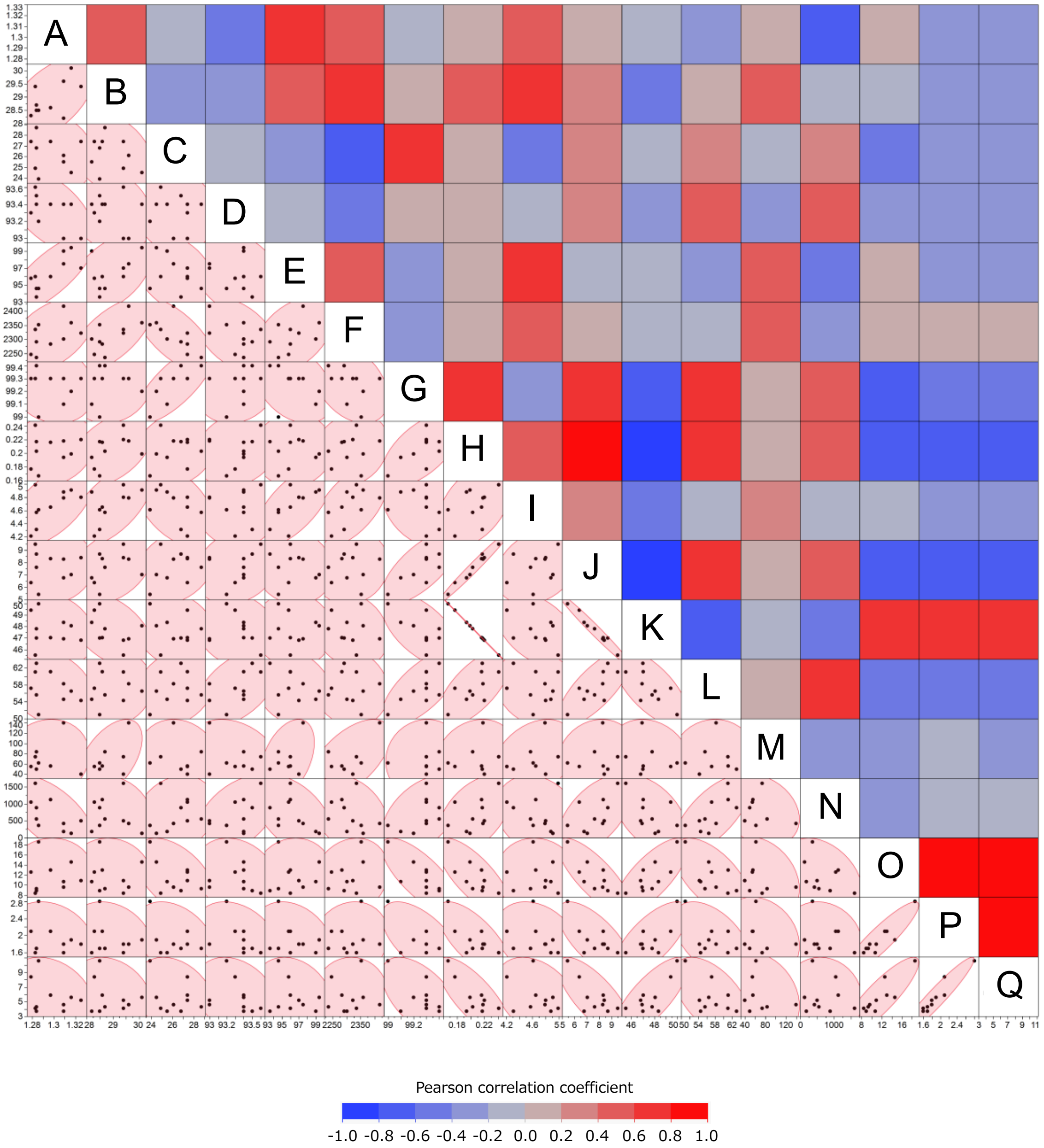

Figure S9. Correlation matrix analysis among the 17 quality attribute (QA) parameters of the 10 products.

The squares arranged on the diagonal line from the top left to the bottom right of the graph show the QA parameters of the 10 products. (A) A260/A280 of the product, (B) Z-average diameter, (C) D90, (D) $s_{20,w}$ of full particle, (E) Full-empty ratio, (F) Nucleic acid length, (G) VP purity, (H) (VP1+VP2)/VP_{total}, (I) Stoichiometric ratio of VP1 molecules, (J) Stoichiometric ratio of VP2 molecules, (K) Stoichiometric ratio of VP3 molecules, (L) Transduction efficiency, (M) HCP, (N) HCD, (O) Deamidation ratio of N57, (P)

Deamidation ratio of N94, (Q) Deamidation ratio of N263. The heatmaps displayed above the diagonal line correspond to Pearson correlation coefficient. The colors of the filled areas are differentiated according to the legend. The correlation plot and the probability ellipse of the 95% confidence interval are shown below the diagonal line.

SUPPLEMENTAL TABLES

Table S1. Comparison of full–empty ratios obtained by cryo-EM and BS-AUC (unit: %).

	Product No.									
	1	2	3	4	5	6	7	8	9	10
Cryo-EM	95.7	97.9*	96.5	94.7	98.2	94.1	93.9	91.2	94.6	95.1
BS-AUC	97.5	99.0	97.0	96.0	99.4	94.6	93.6	94.6	95.8	96.0

*Reported in the previous study.[2]

Table S2. Summary of the deamidation ratio, the stoichiometric ratio of VP2, and the transduction efficiency of Prod. 4, 6, 9, and 10.

Product No.		Deamidation ratio (%) N57	Deamidation ratio (%) N94	Stoichiometric ratio of VP2	Transduction efficiency (%)
4	Average	8.4	1.6	9.5	61.0
	SD	1.1	0.1	0.0	2.2
6	Average	9.3	1.7	7.7	55.6
	SD	0.8	0.1	0.2	1.5
9	Average	12.6	2.1	6.4	57.2
	SD	1.1	0.1	0.0	0.8
10	Average	13.0	2.1	8.3	61.1
	SD	0.2	0.3	0.1	1.1

The deamidation ratios of Prod. 4 and 6 are the same level (Student's t-test p-value≥0.05), whereas the higher VP2 stoichiometric ratio, the higher transduction efficiency. A similar phenomenon was observed in the comparison of Prod. 9 and 10.

REFERENCE

1. Brautigam, C.A. (2015). Calculations and Publication-Quality Illustrations for Analytical Ultracentrifugation Data 1st ed. (Elsevier Inc.) 10.1016/bs.mie.2015.05.001.
2. Nishiumi, H., Hirohata, K., Fukuhara, M., Matsushita, A., Tsunaka, Y., Rocafort, M. allen V., Maruno, T., Torisu, T., and Uchiyama, S. (2024). Combined 100 keV Cryo-Electron Microscopy and Image Analysis Methods to Characterize the Wider Adeno-Associated Viral Products. *113*, 1804–1815. 10.1016/j.xphs.2024.03.026.
